# Tabes Dorsalis and Aluminium Metallicum

**Published:** 1888-10

**Authors:** 


### TABES DORSALIS AND ALUMINIUM METALLICUM.

[Translated from the *Allgemeine Homœopathische Zeitung.*]

"No person sick with this disease," says the learned and experienced Dr. Romberg, speaking of *tabes dorsalis*, in his work upon diseases of the nerve, "*no person sick with this disease can have the faintest hope of recovery; all are doomed.* The only comfort for those who cling to life is in the fact that the disease is of long duration. If in any disease the activity and energy of the physician heightens the malady, it is especially so in tabes dorsalis. You seldom find a patient of this kind without his back full of scars, who has not had volumes of prescriptions, and been at almost all the watering places, in the vain quest of health. It is but the dictate of humanity to tell him that by therapeutical interference his case becomes worse, and that it is only by the strictest care of his *diet*, that the progress of the malady can be stayed."

No further testimony is needed to the incurability of this not unfrequent, but unmistakable and clearly defined disease, although such may be found in all the works on pathology, both new and old. So much the more, may the younger, purely German sister, of the two thousand years old, foreign-born allopathy, congratulate herself, that in accordance with that principle in nature, *similia similibus*, which her founder discovered, she has found a remedy which has been successfully applied in several cases.

The importance of this discovery is sufficient to warrant its being mentioned again ; and as the distance of the residence of the patient of whom I spoke in a former article (and of two others, who were treated in the same manner with the some results) may have created doubts in the minds of skeptics as to the accuracy of the diagnosis, I shall certainly be justified in detailing a recent case which I have had constantly under my own eyes, which I treated with the same remedies, and, as in the other cases, effected a perfect cure.

Miss Frances von W., nineteen years of age, and of one of the first families in Munster—before coming to this city, ten years ago—had been subject to some complaints which exhibited clear symptoms of a chronic (psoric) dyscrasia. At her former place of residence, besides the inevitable Cod liver oil, she had been treated by a homœopathic physician, since deceased, who had given her *Puls.*, *Sulph.*, *Calc. carb.*, *Carb. veg.*, *Sil.*, and *Lyc.*, with only partial success.

The first entry in my journal, under date of December 27th, 1848, is as follows: For four weeks past, an eruption on the head, always moist, especially behind and above both ears, with most severe pains early in the morning, and during the evening. Abdomen, hard and distended. She dislikes meat, and is fond of milk, bread and butter, and all kinds of green vegetables. Sleeps too long, till late in the day. Curvature of the spine, and protruding of the shoulder-blade. Chillblains on her toes, not on her hands. She feels worst in the morning, not so bad in the evening.

To specify all the early treatment of this manifestly scrofulous child would be too lengthy; suffice it to say that the disorder assumed a very obstinate character. The eruption extended not merely over the head and neck, but over the upper portion of the body as far as the genitals; and it was only after the lapse of two years, that the eruption, as well as the curvature of the back, disappeared altogether.

In the years 1851 and 1852, from time to time, there again appeared slight symptoms of disorder, which were readily removed, but which went to show that the inveterate scrofulosis had not been wholly eradicated.

In the spring of 1853 she was attacked with a gastric fever, during which she had a great desire for milk, and on every occasion, she vomited immediately after partaking thereof. She recovered speedily, however, and remained well until the winter of 1853–4, when the eruption, as well as the chillblains on the feet, reappeared, and was not removed until March.

The eruption again made its appearance in the beginning of 1855, but this time lasted only till the middle of February, at which time her courses appeared in great profusion. In the summer of the same year she was attacked with influenza, accompanied by severe pains in the abdomen, which were speedily cured by a dose of Zinc. A long interval followed of apparently perfect health.

During this period she attended a boarding-school four miles distant. While there, in September, 1857, symptoms of disease reappeared. She then complained of severe headache in the evening, with bleeding at the nose; menstruation too copious, and at too short intervals; every motion, every mental or physical exertion, increased her suffering. *Bell.*, *Bry.*, and *Phos.*, each in high potencies, and a single dose, almost entirely removed these complaints; still as sitting in a warm room in the evening seemed to occasion a slight recurrence of the former complaints, I prescribed *Puls.* in the same form with success.

About the middle of January, 1858, a new symptom appeared, of which the teacher could give but very unsatisfactory accounts. She wrote that the patient had severe pains in the back, which were increased by motion, but disappeared at night. On one occasion they had resulted in tetanus. Nothing more was said in the letter. *Nux vom.*$^{200}$, dissolved in water, three times a day, for three days in succession, effected an immediate and continued improvement; but she began to complain of "pains in the pit of the throat, and of difficulty in swallowing," without giving any more definite description. I had her repeat *Nux vom.* dissolved in water the same as before, a spoonful twice a day, morning and evening for six days in succession.

This second prescription of *Nux vom.* was not only without effect, but the pains in the back reappeared, joined to a new symptom, failing of the voice (which was at the worst morning and evening), rendering it impossible for the patient to speak a loud word. I now, for the first time, learned that while using the first remedy, her speaking had daily become more and more difficult; every effort was fatiguing, as if the tongue were lame, and the patient was obliged to take breath at every word, and a little speaking occasioned excessive fatigue.

*Caust.* was prescribed without the slightest effect; but a week later, after prescribing *Sep.* the voice would return for some hours, but still remained weak and low. All other symptoms remained the same, but by the patient's own report, she was "a little better."

On the 3d of February, I sent a dose of *Sulph.*$^{200}$, which had the effect of producing headache (no more definite description), with bleeding at the nose, which got better by sitting up in bed. All other symptoms were lost in the stereotyped phrase "a little better." They nevertheless still existed, but nothing more was said by which I could be guided to the choice of the adequate remedies.

Under these perplexing circumstances, on the 17th of

February, I sent a dose of *Bell.* 200, to be taken as before, and requested her parents to send for her, as the distance was not very great, and her disease was of a chronic character, so that she could venture home without danger, for I wished to give the case a personal examination. It is worthy of note that among patients of the higher classes of society, especially when the disease is of a nervous character, the physician frequently can hear only the stereotyped phrase "a little better," when in fact there has not been the slightest improvement, but on the other hand new symptoms have appeared, which make the case more complicated. We are thus left without any clue to the selection of the remedial agent.

In compliance with my earnest request, my patient arrived on the 24th of February, and in the evening I went to see her. How great was my surprise, when I discovered that here was a perfect case of tabes dorsalis; for the letters I had received had not given the slightest hint that such was the fact. The loss of voice, on which most stress had been laid, is only an occasional symptom in cases of this disease; and as I had not heard a syllable of the palsy of the lower extremities, which was considerably advanced, I had, of course, been unable to recognize the malady.

When I saw the patient her voice was certainly almost entirely gone, and her utterance was so indistinct that I had to approach my ear closely to her mouth in order to catch the words. But all the other symptoms were too distinct for me to mistake the nature of the disease; and the loss of voice went only to show to what an extent the affection of the spinal marrow must have proceeded.

What I gathered on this first visit was substantially as follows: Sometime previous the patient had experienced great weakness in the lower extremities, accompanied more or less with pains in the back. The sensation in her back was like burning, as if a hot iron rod was being pushed up through the spinal column. In the earlier stages of the malady the feeling had sometimes been that of insects crawling upward. At the same time the soles of her feet had appeared like soft cushions, or as if her feet were placed on a soft blanket or cushion. By and by she lost all sensation in her feet, so that she did not feel the floor on which she stood, and would not have known that she stood upon the floor had she not seen it. For the few last weeks she had been unable to walk; and for sometime previous she could do so only in broad daylight; with her eyes closed, or in the dark, she had stumbled so that she had been obliged to hold on to something to keep from falling. At present she could not, even in daylight, stand on her feet without support. When in bed she had no knowledge of the position of her lower extremities, which assume various strange positions, without any sensation on her part. Before she lost the power of walking when she attempted to walk a few steps in the dark, in rooms that were well known to her, she had always turned to the left, and thus missed her aim. She frequently had a sensation of contraction in the abdomen, as if a band were drawn tightly around it; this feeling, as well as the pains in the back, was more severe, when attempting to move, after resting for some time. Although there were no pains in the throat, the exertion required by her loss of voice, occasioned great exhaustion, so that she was obliged to rest several times during her recital of her symptoms.

In other respects I found the patient looking very well—in good flesh—blooming complexion—complaining very little, and not considering her case alarming, or of much consequence. Appetite and digestion good; passages a little slow and hard. The menses appeared regularly but rather copious. She feels rather better in the morning than in the evening.

The above complete and faithfully recorded symptoms were particularly interesting to me, as they were the first which occurred under my own observation, after my study of *Alumina.* They left no doubt on my mind that this was a striking case of genuine tabes dorsalis, and in accordance with my former experience I did not hesitate to administer at once a dose of *Aluminium metallicum*$^{200}$ (from the Pharmacy of *Lehrmann* in Schoningen). I ordered the medicine to be dissolved in six tablespoonfuls of water, and a spoonful of the solution to be taken three times a day for two days.

The next time I saw the patient, on the 26th of February, a decided improvement had taken place, and I allowed the action of the medicine to continue without interference. On the 1st of March I again left *Alum. met.*$^{200}$, to be taken as before, and the improvement still continued, and as her courses had come on, I delayed a third dose until the 5th of March. My journal mentions a continual improvement in the case; patient is up the whole day, and walks about the house; she even goes up-stairs without much difficulty. But when I caused her to close her eyes and try to walk straight, she invariably turned to the left; nor could she walk in the dark without support.

On the 10th of March, *Alum. met.* as before. The difficulty in the lower extremities is decidedly better, but the voice often fails, especially in the evening, and her utterance is difficult and fatiguing. It is often the case, especially in chronic diseases, that the repetition of a single remedy, without alternation, does not effect a sufficiently rapid cure, and although the symptoms become less violent, they do not essentially change. As this seemed to be the case in the present instance, I administered on the 15th of March, *Nat. mur.*$^{200}$, to be taken in the same manner as the *Alum. met.* The effect was good, but not so decided as from the first remedy. It may be that there is too great similarity between the effects of *Nat. mur.* and of *Alum. met.;* as is often observed, if remedies too nearly related (as *Ign.*, *Nux vom.* and *Puls.*) are given in alternation. Still the improvement continued, so that on the 21st of March the patient came to see me at my office. I gave her *Alumina*$^{3000}$ (Jenichen's) after which the improvement went on more rapidly; but the pains in back increased a little, showing that the effects of this were not so specific as those of *Alum. met.*

On the 28th of March I gave *Caust.*$^{200}$, after which the pains in the back disappeared, and the voice and speaking improved, but at the same time the weakness of the legs and the feeling of numbness in the soles of the feet reappeared. I, therefore, concluded that this remedy does not exactly meet the case of tabes dorsalis. On the 11th of April, *Alum. met.*$^{200}$ again. With this the symptoms almost entirely disappeared, and even her voice assumed its usual sound and the freedom of utterance that belonged to her days of health. As the sensation of crawling in the back recurred now and then, and there was occasionally the sensation of transient numbness in the soles of the feet, on the 20th day of April I administered another dose of *Alum. met.*$^{200}$. On the 28th of April I gave a dose of *Puls.*$^{200}$, and finally, on the 7th of May, a dose of *Sulph.*$^{200}$, after which the last symptoms of the disease disappeared, and nothing of the kind has since recurred.

I watched this case with great interest, and from the above detailed statement the energetic, almost specific efficacy of *Alum. met.* in cases of tabes dorsalis, appears unmistakable, and I do not see what objections can be raised against it. As, for a number of years, I have habitually treated all cases with only the high dynamizations, in the most refined doses, I cannot assert that the lower potencies of this remedy (as of many others, which the old school repudiates, as indifferent or ineffectual) may not be of as much, or even more, effect in this case. The fact that I accomplished more rapid and certain cures, even in acute diseases, with high potencies, which I have used since 1843, than I formerly did with the lower dynamizations, can hardly be deemed conclusive; for by my larger practice, and more thorough acquaintance with the nature of our drugs, I have acquired a greater facility in the selection of the remedy. But this I consider as certain, that it is not only possible to effect cures in the most difficult cases, with high potencies, but that such cures, especially in chronic cases, are more thorough and lasting than those effected by lower dilutions. I trust that some of my colleagues who prefer the latter will try *Alum. met.* in this disease, which has so clear a diagnosis, and which is entirely beyond the reach of allopathy. For, as Hahnemann has shown with *Merc.* and *Thuja*, it is only in individual diseases, and with specific remedies that we can decide between the higher and lower potencies, and it is only under such circumstances that experience can be considered conclusive.

It may not be superfluous to mention here that of late years, especially in France, under the guidance of a learned and highly gifted man a school has grown up, which, although claiming to belong to the homœopathic, questions the validity of symptoms observed in the healthy, or, in other words, denies the law of "similia similibus" to be a principle of nature. Still the discovery of this remedy for tabes dorsalis was the result of the study of *Alumina* (in Hahnemann's *Chronic Diseases*), for this is the only remedy among those which have thus far been proved that has the most striking and characteristic symptoms of this disease. Of course it must be admitted that the provings of *Alumina* are far from perfect—that a great deal is left indefinite—that the effects of changes and after effects are not clearly defined, and that much remains to be ascertained by careful practice as to the true value and clear definition of the drug. Physicians should give their especial attention to the recognition and pointing out of those incidental and deceitful results which render the study of the different remedies so difficult.

I have reason to believe that I have in this way discovered the most effectual remedy for diabetes mellitus, but I forbear to give it publicity until renewed trials have overcome all uncertainty about the matter.—*Bœnninghausen.*

